# Sodium *N*,2-dichloro­benzene­sulfonamidate sesquihydrate

**DOI:** 10.1107/S1600536810009864

**Published:** 2010-03-20

**Authors:** B. Thimme Gowda, Sabine Foro, K. Shakuntala, Hartmut Fuess

**Affiliations:** a Department of Chemistry, Mangalore University, Mangalagangotri 574 199, Mangalore, India; b Institute of Materials Science, Darmstadt University of Technology, Petersenstrasse 23, D-64287 Darmstadt, Germany

## Abstract

In the title compound, Na^+^·C_6_H_4_Cl_2_NO_2_S^−^·1.5H_2_O, one of the water mol­ecules lies on a twofold axis. There is no inter­action between the N atom and the sodium ion. The sodium ion exhibits a pseudo-octa­hedral coordination defined by three water O atoms and three sulfonyl O atoms from three different anions. The S—N distance of 1.588 (2) Å is consistent with an S=N double bond. The crystal structure is stabilized by O—H⋯N and O—H⋯Cl hydrogen bonds.

## Related literature

For background to *N*-haloaryl­sulfonamides, see: Gowda *et al.* (2005 ▶). For related structures, see: Gowda *et al.* (2007 ▶, 2009 ▶); George *et al.* (2000 ▶); Olmstead & Power (1986 ▶).
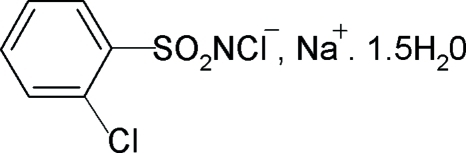


## Experimental

### 

#### Crystal data

Na^+^·C_6_H_4_Cl_2_NO_2_S^−^·1.5H_2_O
*M* *_r_* = 275.08
Monoclinic, 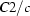

*a* = 11.1288 (7) Å
*b* = 6.6724 (4) Å
*c* = 28.144 (2) Å
β = 102.274 (6)°
*V* = 2042.1 (2) Å^3^
*Z* = 8
Mo *K*α radiation
μ = 0.87 mm^−1^
*T* = 299 K
0.46 × 0.36 × 0.28 mm

#### Data collection

Oxford Diffraction Xcalibur diffractometer with a Sapphire CCD detector
Absorption correction: multi-scan (*CrysAlis RED*; Oxford Diffraction, 2009 ▶) *T*_min_ = 0.691, *T*_max_ = 0.794
6590 measured reflections
2076 independent reflections
1944 reflections with *I* > 2σ(*I*)
*R*_int_ = 0.014

#### Refinement

*R*[*F* ^2^ > 2σ(*F* ^2^)] = 0.029
*wR*(*F* ^2^) = 0.069
*S* = 1.15
2076 reflections
141 parameters
3 restraints
H atoms treated by a mixture of independent and constrained refinement
Δρ_max_ = 0.36 e Å^−3^
Δρ_min_ = −0.28 e Å^−3^

### 

Data collection: *CrysAlis CCD* (Oxford Diffraction, 2009 ▶); cell refinement: *CrysAlis RED* (Oxford Diffraction, 2009 ▶); data reduction: *CrysAlis RED*; program(s) used to solve structure: *SHELXS97* (Sheldrick, 2008 ▶); program(s) used to refine structure: *SHELXL97* (Sheldrick, 2008 ▶); molecular graphics: *PLATON* (Spek, 2009 ▶); software used to prepare material for publication: *SHELXL97*.

## Supplementary Material

Crystal structure: contains datablocks I, global. DOI: 10.1107/S1600536810009864/bx2268sup1.cif

Structure factors: contains datablocks I. DOI: 10.1107/S1600536810009864/bx2268Isup2.hkl

Additional supplementary materials:  crystallographic information; 3D view; checkCIF report

## Figures and Tables

**Table 1:** Hydrogen-bond geometry (Å, °)

*D*—H⋯*A*	*D*—H	H⋯*A*	*D*⋯*A*	*D*—H⋯*A*
O3—H31⋯N1^i^	0.79 (2)	2.15 (2)	2.926 (2)	166 (3)
O3—H32⋯Cl1^ii^	0.81 (2)	2.67 (2)	3.4782 (16)	171 (2)
O4—H41⋯N1^ii^	0.81 (2)	2.19 (2)	3.005 (2)	176 (2)

Symmetry codes: (i) 
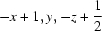
; (ii) 
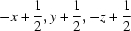
.

**Fig. 1.**
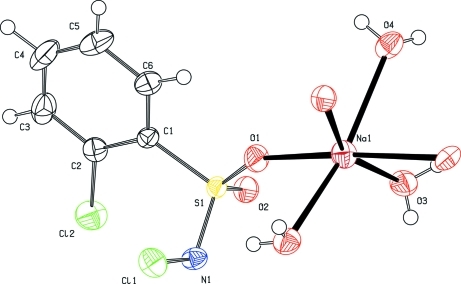
Molecular structure of the title compound, showing the atom labelling scheme. The displacement ellipsoids are drawn at the 50% probability level. The H atoms are represented as small spheres of arbitrary radii.

**Fig. 2.**
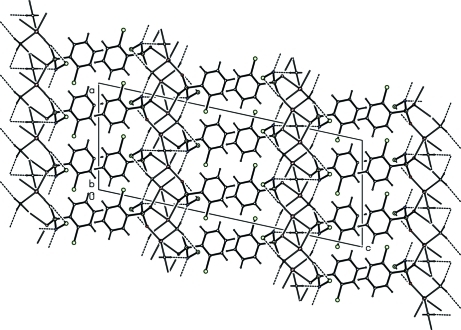
Molecular packing of the title compound with hydrogen bonding shown as dashed lines.

## supplementary crystallographic information

### Comment

In the present work, as a part of exploring the substituent effects on the solid-state structures of N-halo arylsulfonamidates (Gowda et al., 2005; 2007; 2009), the structure of sodium N-chloro-2-chloro- benzenesulfonamidate (I) has been determined (Fig. 1). The structure of (I) resembles the sodium salts of N-chloro-4-chlorobenzenesulfonamidate (Gowda et al., 2007), N-chloro-2-methylbenzenesulfonamidate (Gowda et al., 2009), and other sodium N-chloro- arylsulfonamidates (George et al., 2000; Olmstead & Power, 1986).

The sodium ion shows pseudo-octahedral coordination defined by three water-O atoms and by three sulfonyl-O atoms derived from three different anions. There is no interaction between the nitrogen and sodium ions. The S—N distance of 1.588 (2)Å is consistent with a S—N double bond and is in agreement with those observed with related N-chloro arylsulfonamides.

The Packing diagram consists of a two-dimensional polymeric layer running parallel to the ac plane (Fig. 2). The molecular packing is stabilized by N-H···O and O-H···Cl hydrogen bonds (Table 1)

### Experimental

The title compound was prepared according to the literature method (Gowda *et al.*, 2005; 2007). The purity of the compound was checked by determining its melting point. It was characterized by recording its infrared and NMR spectra. Single crystals of the title compound used in X-ray diffraction studies were obtained from a slow evaporation of its chloroform solution at room temperature.

### Refinement

The O-bound H atoms were located in difference map and later restrained to O—H = 0.82 (2) Å. The other H atoms were positioned with idealized geometry using a riding model with C—H = 0.93 Å. All H atoms were refined with isotropic displacement parameters (set to 1.2 times of the *U*_eq_ of the parent atom).

### Crystal data

**Table:**

Na^+^·C_6_H_4_Cl_2_NO_2_S^−^·1.5H_2_O	*F*(000) = 1112
*M**_r_* = 275.08	*D*_x_ = 1.789 Mg m^−^^3^
Monoclinic, *C*2/*c*	Mo *K*α radiation, λ = 0.71073 Å
Hall symbol: -C 2yc	Cell parameters from 2816 reflections
*a* = 11.1288 (7) Å	θ = 3.0–27.9°
*b* = 6.6724 (4) Å	µ = 0.87 mm^−^^1^
*c* = 28.144 (2) Å	*T* = 299 K
β = 102.274 (6)°	Prism, colourless
*V* = 2042.1 (2) Å^3^	0.46 × 0.36 × 0.28 mm
*Z* = 8	

### Data collection

**Table:**

Oxford Diffraction Xcalibur diffractometer with a Sapphire CCD detector	2076 independent reflections
Radiation source: fine-focus sealed tube	1944 reflections with *I* > 2σ(*I*)
graphite	*R*_int_ = 0.014
Rotation method data acquisition using ω and phi scans	θ_max_ = 26.4°, θ_min_ = 3.0°
Absorption correction: multi-scan (*CrysAlis RED*; Oxford Diffraction, 2009)	*h* = −13→13
*T*_min_ = 0.691, *T*_max_ = 0.794	*k* = −8→6
6590 measured reflections	*l* = −33→35

### Refinement

**Table:**

Refinement on *F*^2^	Primary atom site location: structure-invariant direct methods
Least-squares matrix: full	Secondary atom site location: difference Fourier map
*R*[*F*^2^ > 2σ(*F*^2^)] = 0.029	Hydrogen site location: inferred from neighbouring sites
*wR*(*F*^2^) = 0.069	H atoms treated by a mixture of independent and constrained refinement
*S* = 1.15	*w* = 1/[σ^2^(*F*_o_^2^) + (0.0246*P*)^2^ + 3.4504*P*] where *P* = (*F*_o_^2^ + 2*F*_c_^2^)/3
2076 reflections	(Δ/σ)_max_ = 0.006
141 parameters	Δρ_max_ = 0.36 e Å^−^^3^
3 restraints	Δρ_min_ = −0.28 e Å^−^^3^

### Special details

**Table:**

Experimental. (CrysAlis RED; Oxford Diffraction, 2009) Empirical absorption correction using spherical harmonics, implemented in SCALE3 ABSPACK scaling algorithm.
Geometry. All esds (except the esd in the dihedral angle between two l.s. planes) are estimated using the full covariance matrix. The cell esds are taken into account individually in the estimation of esds in distances, angles and torsion angles; correlations between esds in cell parameters are only used when they are defined by crystal symmetry. An approximate (isotropic) treatment of cell esds is used for estimating esds involving l.s. planes.
Refinement. Refinement of *F*^2^ against ALL reflections. The weighted *R*-factor wR and goodness of fit *S* are based on *F*^2^, conventional *R*-factors *R* are based on *F*, with *F* set to zero for negative *F*^2^. The threshold expression of *F*^2^ > σ(*F*^2^) is used only for calculating *R*-factors(gt) etc. and is not relevant to the choice of reflections for refinement. *R*-factors based on *F*^2^ are statistically about twice as large as those based on *F*, and *R*- factors based on ALL data will be even larger.

### Fractional atomic coordinates and isotropic or equivalent isotropic displacement parameters (Å^2^)

**Table:**

	*x*	*y*	*z*	*U*_iso_*/*U*_eq_	
C1	0.31355 (16)	0.8280 (3)	0.10792 (6)	0.0208 (4)	
C2	0.39097 (18)	0.8966 (3)	0.07759 (7)	0.0257 (4)	
C3	0.3434 (2)	0.9809 (3)	0.03289 (8)	0.0376 (5)	
H3	0.3951	1.0261	0.0132	0.045*	
C4	0.2181 (2)	0.9965 (4)	0.01810 (8)	0.0454 (6)	
H4	0.1843	1.0514	−0.0122	0.054*	
C5	0.1413 (2)	0.9312 (4)	0.04792 (9)	0.0431 (6)	
H5	0.0566	0.9446	0.0375	0.052*	
C6	0.18813 (18)	0.8472 (3)	0.09260 (7)	0.0301 (4)	
H6	0.1358	0.8038	0.1122	0.036*	
Cl1	0.38325 (5)	0.35684 (8)	0.123234 (19)	0.03468 (14)	
Cl2	0.54842 (5)	0.88282 (9)	0.09392 (2)	0.04180 (16)	
N1	0.45784 (14)	0.5415 (2)	0.16212 (6)	0.0255 (3)	
Na1	0.14395 (7)	0.50352 (13)	0.23560 (3)	0.03065 (19)	
O1	0.25555 (13)	0.6627 (2)	0.18286 (5)	0.0329 (3)	
O2	0.44039 (12)	0.8680 (2)	0.19636 (5)	0.0295 (3)	
O3	0.29191 (13)	0.6793 (2)	0.29703 (5)	0.0333 (3)	
H31	0.3538 (18)	0.627 (4)	0.3106 (9)	0.040*	
H32	0.258 (2)	0.718 (4)	0.3182 (8)	0.040*	
O4	0.0000	0.7742 (3)	0.2500	0.0336 (5)	
H41	0.013 (2)	0.852 (3)	0.2729 (7)	0.040*	
S1	0.36626 (4)	0.71995 (7)	0.166416 (15)	0.02023 (12)	

### Atomic displacement parameters (Å^2^)

**Table:**

	*U*^11^	*U*^22^	*U*^33^	*U*^12^	*U*^13^	*U*^23^
C1	0.0244 (9)	0.0166 (8)	0.0201 (8)	0.0008 (7)	0.0022 (7)	−0.0003 (7)
C2	0.0290 (10)	0.0207 (9)	0.0277 (9)	−0.0013 (8)	0.0068 (8)	−0.0009 (8)
C3	0.0540 (14)	0.0311 (11)	0.0293 (11)	−0.0020 (10)	0.0129 (10)	0.0059 (9)
C4	0.0605 (15)	0.0406 (13)	0.0279 (11)	0.0060 (12)	−0.0068 (10)	0.0108 (10)
C5	0.0355 (12)	0.0442 (13)	0.0412 (12)	0.0054 (10)	−0.0106 (9)	0.0065 (11)
C6	0.0239 (9)	0.0331 (11)	0.0310 (10)	0.0007 (8)	0.0008 (8)	0.0010 (9)
Cl1	0.0399 (3)	0.0266 (3)	0.0380 (3)	−0.0038 (2)	0.0092 (2)	−0.0080 (2)
Cl2	0.0281 (3)	0.0487 (3)	0.0520 (3)	−0.0023 (2)	0.0163 (2)	0.0120 (3)
N1	0.0243 (8)	0.0226 (8)	0.0269 (8)	0.0010 (7)	−0.0002 (6)	−0.0014 (7)
Na1	0.0281 (4)	0.0325 (4)	0.0328 (4)	−0.0052 (3)	0.0097 (3)	0.0006 (3)
O1	0.0272 (7)	0.0430 (9)	0.0309 (7)	−0.0009 (6)	0.0116 (6)	0.0065 (7)
O2	0.0295 (7)	0.0308 (8)	0.0252 (7)	−0.0014 (6)	−0.0007 (5)	−0.0081 (6)
O3	0.0245 (7)	0.0408 (9)	0.0333 (8)	0.0014 (7)	0.0035 (6)	−0.0031 (7)
O4	0.0432 (12)	0.0244 (11)	0.0291 (11)	0.000	−0.0015 (9)	0.000
S1	0.0197 (2)	0.0233 (2)	0.0173 (2)	−0.00026 (17)	0.00302 (15)	0.00009 (17)

### Geometric parameters (Å, °)

**Table:**

C1—C6	1.376 (3)	Na1—O2^i^	2.4710 (15)
C1—C2	1.412 (3)	Na1—O2^ii^	2.4759 (15)
C1—S1	1.7786 (18)	Na1—O4	2.5035 (18)
C2—C3	1.377 (3)	Na1—O3^ii^	2.5120 (18)
C2—Cl2	1.717 (2)	Na1—S1^ii^	3.3661 (9)
C3—C4	1.372 (3)	O1—S1	1.4562 (14)
C3—H3	0.9300	O2—S1	1.4390 (14)
C4—C5	1.389 (4)	O2—Na1^iii^	2.4710 (15)
C4—H4	0.9300	O2—Na1^iv^	2.4759 (15)
C5—C6	1.374 (3)	O3—Na1^iv^	2.5120 (18)
C5—H5	0.9300	O3—H31	0.792 (16)
C6—H6	0.9300	O3—H32	0.811 (16)
Cl1—N1	1.7376 (16)	O4—Na1^v^	2.5035 (18)
N1—S1	1.5883 (16)	O4—H41	0.814 (16)
Na1—O1	2.3785 (15)	S1—Na1^iv^	3.3661 (9)
Na1—O3	2.4220 (17)		
			
C6—C1—C2	119.28 (17)	O2^ii^—Na1—O3^ii^	98.75 (6)
C6—C1—S1	116.14 (14)	O4—Na1—O3^ii^	157.17 (5)
C2—C1—S1	124.58 (14)	O1—Na1—S1^ii^	151.07 (5)
C3—C2—C1	121.30 (19)	O3—Na1—S1^ii^	79.85 (4)
C3—C2—Cl2	116.02 (16)	O2^i^—Na1—S1^ii^	88.45 (4)
C1—C2—Cl2	122.68 (15)	O2^ii^—Na1—S1^ii^	22.58 (3)
C4—C3—C2	118.5 (2)	O4—Na1—S1^ii^	97.97 (3)
C4—C3—H3	120.8	O3^ii^—Na1—S1^ii^	82.95 (4)
C2—C3—H3	120.8	S1—O1—Na1	154.80 (9)
C3—C4—C5	120.6 (2)	S1—O2—Na1^iii^	150.45 (9)
C3—C4—H4	119.7	S1—O2—Na1^iv^	116.06 (8)
C5—C4—H4	119.7	Na1^iii^—O2—Na1^iv^	89.02 (5)
C6—C5—C4	121.3 (2)	Na1—O3—Na1^iv^	111.04 (6)
C6—C5—H5	119.4	Na1—O3—H31	121.4 (19)
C4—C5—H5	119.4	Na1^iv^—O3—H31	105.4 (19)
C5—C6—C1	119.1 (2)	Na1—O3—H32	108.9 (19)
C5—C6—H6	120.5	Na1^iv^—O3—H32	102.1 (19)
C1—C6—H6	120.5	H31—O3—H32	106 (3)
S1—N1—Cl1	110.56 (9)	Na1^v^—O4—Na1	87.67 (8)
O1—Na1—O3	82.14 (6)	Na1^v^—O4—H41	109.7 (18)
O1—Na1—O2^i^	115.80 (6)	Na1—O4—H41	125.3 (18)
O3—Na1—O2^i^	156.33 (6)	O2—S1—O1	114.36 (9)
O1—Na1—O2^ii^	168.48 (6)	O2—S1—N1	105.17 (8)
O3—Na1—O2^ii^	86.38 (6)	O1—S1—N1	115.30 (9)
O2^i^—Na1—O2^ii^	75.50 (6)	O2—S1—C1	107.39 (9)
O1—Na1—O4	102.47 (6)	O1—S1—C1	105.41 (8)
O3—Na1—O4	84.05 (5)	N1—S1—C1	108.91 (8)
O2^i^—Na1—O4	77.23 (5)	O2—S1—Na1^iv^	41.36 (6)
O2^ii^—Na1—O4	77.14 (5)	O1—S1—Na1^iv^	73.08 (6)
O1—Na1—O3^ii^	85.97 (6)	N1—S1—Na1^iv^	128.14 (6)
O3—Na1—O3^ii^	118.37 (5)	C1—S1—Na1^iv^	117.93 (6)
O2^i^—Na1—O3^ii^	79.99 (5)		
			
C6—C1—C2—C3	−0.5 (3)	O2^ii^—Na1—O4—Na1^v^	−38.86 (3)
S1—C1—C2—C3	−179.26 (16)	O3^ii^—Na1—O4—Na1^v^	43.09 (13)
C6—C1—C2—Cl2	178.96 (16)	S1^ii^—Na1—O4—Na1^v^	−47.647 (17)
S1—C1—C2—Cl2	0.2 (2)	Na1^iii^—O2—S1—O1	141.67 (17)
C1—C2—C3—C4	−0.2 (3)	Na1^iv^—O2—S1—O1	−3.82 (12)
Cl2—C2—C3—C4	−179.70 (18)	Na1^iii^—O2—S1—N1	14.2 (2)
C2—C3—C4—C5	0.9 (4)	Na1^iv^—O2—S1—N1	−131.33 (9)
C3—C4—C5—C6	−0.8 (4)	Na1^iii^—O2—S1—C1	−101.75 (18)
C4—C5—C6—C1	0.1 (4)	Na1^iv^—O2—S1—C1	112.77 (9)
C2—C1—C6—C5	0.5 (3)	Na1^iii^—O2—S1—Na1^iv^	145.5 (2)
S1—C1—C6—C5	179.42 (17)	Na1—O1—S1—O2	−71.7 (2)
O3—Na1—O1—S1	50.3 (2)	Na1—O1—S1—N1	50.4 (3)
O2^i^—Na1—O1—S1	−145.9 (2)	Na1—O1—S1—C1	170.6 (2)
O2^ii^—Na1—O1—S1	45.6 (5)	Na1—O1—S1—Na1^iv^	−74.3 (2)
O4—Na1—O1—S1	132.4 (2)	Cl1—N1—S1—O2	−175.92 (9)
O3^ii^—Na1—O1—S1	−69.0 (2)	Cl1—N1—S1—O1	57.14 (12)
S1^ii^—Na1—O1—S1	−1.5 (3)	Cl1—N1—S1—C1	−61.07 (11)
O1—Na1—O3—Na1^iv^	31.28 (6)	Cl1—N1—S1—Na1^iv^	144.96 (6)
O2^i^—Na1—O3—Na1^iv^	−109.93 (14)	C6—C1—S1—O2	−118.21 (15)
O2^ii^—Na1—O3—Na1^iv^	−149.66 (7)	C2—C1—S1—O2	60.61 (18)
O4—Na1—O3—Na1^iv^	−72.23 (6)	C6—C1—S1—O1	4.11 (17)
O3^ii^—Na1—O3—Na1^iv^	112.35 (9)	C2—C1—S1—O1	−177.07 (16)
S1^ii^—Na1—O3—Na1^iv^	−171.45 (6)	C6—C1—S1—N1	128.38 (15)
O1—Na1—O4—Na1^v^	152.95 (5)	C2—C1—S1—N1	−52.80 (18)
O3—Na1—O4—Na1^v^	−126.49 (5)	C6—C1—S1—Na1^iv^	−74.61 (16)
O2^i^—Na1—O4—Na1^v^	38.93 (4)	C2—C1—S1—Na1^iv^	104.21 (15)

Symmetry codes: (i) *x*−1/2, *y*−1/2, *z*; (ii) −*x*+1/2, *y*−1/2, −*z*+1/2; (iii) *x*+1/2, *y*+1/2, *z*; (iv) −*x*+1/2, *y*+1/2, −*z*+1/2; (v) −*x*, *y*, −*z*+1/2.

### Hydrogen-bond geometry (Å, °)

**Table:**

*D*—H···*A*	*D*—H	H···*A*	*D*···*A*	*D*—H···*A*
O3—H31···N1^vi^	0.79 (2)	2.15 (2)	2.926 (2)	166 (3)
O3—H32···Cl1^iv^	0.81 (2)	2.67 (2)	3.4782 (16)	171 (2)
O4—H41···N1^iv^	0.81 (2)	2.19 (2)	3.005 (2)	176 (2)

Symmetry codes: (vi) −*x*+1, *y*, −*z*+1/2; (iv) −*x*+1/2, *y*+1/2, −*z*+1/2.
